# The Lack of TRPA1 Ion Channel Does Not Affect the Chronic Stress-Induced Activation of the Locus Ceruleus

**DOI:** 10.3390/ijms25031765

**Published:** 2024-02-01

**Authors:** Milica Milicic, Balázs Gaszner, Gergely Berta, Erika Pintér, Viktória Kormos

**Affiliations:** 1 Department of Pharmacology and Pharmacotherapy, Medical School, University of Pécs, H-7624 Pécs, Hungary; milicamilicic97@live.no (M.M.); erika.pinter@aok.pte.hu (E.P.); 2 Department of Anatomy, Medical School and Research Group for Mood Disorders, University of Pécs, H-7624 Pécs, Hungary; balazs.b.gaszner@aok.pte.hu; 3 Department of Medical Biology, Medical School, University of Pécs, H-7624 Pécs, Hungary; gergely.berta@aok.pte.hu

**Keywords:** TRPA1, stress, CVMS, depression

## Abstract

We have previously proven the involvement of transient receptor potential ankyrin 1 (TRPA1) in stress adaptation. A lack of TRPA1 affects both urocortin 1 (member of the corticotropin-releasing hormone (CRH) family) content of the Edinger–Westphal nucleus. The noradrenergic locus ceruleus (LC) is also an important player in mood control. We aimed at investigating whether the TRPA1 is expressed in the LC, and to test if the response to chronic variable mild stress (CVMS) is affected by a lack of TRPA1. The TRPA1 expression was examined via RNAscope in situ hybridization. We investigated TRPA1 knockout and wildtype mice using the CVMS model of depression. Tyrosine hydroxylase (TH) and FOSB double immunofluorescence were used to test the functional neuromorphological changes in the LC. No TRPA1 expression was detected in the LC. The TH content was not affected by CVMS exposure. The CVMS-induced FOSB immunosignal did not co-localize with the TH neurons. TRPA1 is not expressed in the LC. A lack of functional TRPA1 receptor neither directly nor indirectly affects the TH content of LC neurons under CVMS.

## 1. Introduction

Transient receptor potential ankyrin 1 (TRPA1) is a non-selective cation channel whose role in the peripheral nervous system (PNS) is well known, involving primarily nociception, as well as inflammatory and immune responses [1,2,3,4,5]. TRPA1 was shown to be predominantly expressed by primary sensory neurons of the dorsal root, vagal and trigeminal ganglia [6]. It is activated by painful cold [6,7], heat and mechanical stimuli [4], as well as reactive ligands (e.g., hydrogen peroxide, hydrogen sulfide, cyclopentenone prostaglandin, nitro-oleic acid, 4-oxononenal and reactive oxygen species) produced during oxidative stress, neuroinflammation and degenerative processes [3,8]. Upon activation, the calcium influx triggers several intracellular pathways [9].

Despite the widespread knowledge and agreement about the function of TRPA1 in the PNS, our knowledge about its role and distribution in the central nervous system (CNS) remains insufficient. We confirmed the presence of its mRNA via highly sensitive and specific RNAscope in situ hybridization (ISH) in certain stress-related limbic brain areas, including the olfactory bulb, piriform cortex [10] and hypothalamus [11]. Nevertheless, the highest level of *Trpa1* mRNA expression was detected in the urocortinergic cells of the centrally projecting Edinger–Westphal nucleus (EWcp) [12].

*Trpa1* knockout (KO) mice were previously shown to exhibit a reduced response to specific acute and chronic TRPA1-mediated nociceptive stimuli [7,13,14,15,16,17], which can be attributed to functional changes in the sensory neurons of the PNS [18]. However, there is currently limited information available about the potential role of TRPA1 receptors in long-term complex central adaptation responses, such as in adaptation to chronic stress. The *Trpa1* mRNA expression was confirmed by our research group in both the mouse and human EWcp, which was downregulated in both mice exposed to the chronic variable mild stress (CVMS) model of depression and in depressed human suicide victims [12]. Moreover, an altered stress adaptation capacity was observed in *Trpa1* gene-deficient mice in the single prolonged stress model of posttraumatic stress disorder (PTSD) [19]. These findings suggest that TRPA1 in EWcp/urocortinergic neurons might contribute to the regulation of depression-like behavior and the stress adaptation response in mice [12].

The prevalence of stress-related disorders has been steadily rising over the last years, which emphasizes the importance of research in this area. The two major limbs of stress adaptation include the hypothalamic–pituitary–adrenal (HPA) axis controlled by the paraventricular nucleus (PVN) of the hypothalamus, and the sympatho-adrenomedullary noradrenergic system [20]. The locus ceruleus (LC), localized in the floor of the fourth ventricle within the dorsolateral pontine tegmentum, is the primary source of noradrenaline (NA) in the CNS. To examine the role of TRPA1 in mood regulation in the noradrenergic system, our obvious choice of target for this experiment was the LC, known for its vast and divergent efferent system, whose noradrenergic fibers reach nearly the entire neuroaxis. This system is mainly involved in the fast physiological responses to fear, stress and panic [20].

Although the LC is more known for its role in acute stress, the noradrenergic system contributes also to the chronic stress response [21,22,23]. The central noradrenergic system is also implicated in emotion control; the chronic stress-induced dysregulation of the noradrenergic system was shown to play a role in the pathogenesis of affective disorders, especially depression [23,24]. LC noradrenergic neurons were shown to exhibit hypoactivity in both a rat model of depression [25] and in the human [26]. A decreased level of monoamines in the brain has long-time been believed to be the main causative factor of depression [27], and many antidepressants used today work by increasing the monoamine level in the synapses of the brain. Fast-acting antidepressants also suppress the upregulation of noradrenergic neurotransmission by LC neurons [23]. Furthermore, the symptoms and pathophysiology of other psychiatric conditions, such as panic disorder and PTSD, have been associated with alternations in the noradrenergic system [21,28]. Moreover, lower levels of an NA metabolite in the cerebrospinal fluid were associated with increased suicide risk [29].

One of the known targets of LC neurons is the PVN of the hypothalamus. In response to stress, the NA release stimulates the noradrenergic receptors in the corticotropin-releasing hormone (CRH)-containing neurons of the PVN [23,30]. On the other hand, CRH neurons, mainly from the PVN, directly control LC neuronal excitability and activity via CRH receptor 1(CRH-R1), meaning that the connection between the LC and PVN is bidirectional [30].

Primarily, we aimed at testing whether the *Trpa1* is expressed in the LC. Secondly, we proposed to investigate if the response to the CVMS model of depression is affected by a lack of functional TRPA1. This was examined through a comparison of tyrosine hydroxylase (TH) content in the LC between *Trpa1* KO and WT mice upon chronic stress. We used TH as it is the rate-limiting enzyme of NA biosynthesis, and the assessment of its level allows for a reliable evaluation of the dynamics of NA synthesis. The CVMS-induced neuronal activation of the LC was evaluated in *Trpa1* KO vs. WT mice. For this purpose, we used FOSB, a reliable marker of chronic neuronal activation.

## 2. Results

### 2.1. Examination of Trpa1 Expression in the Locus Ceruleus

**Figure 1:**
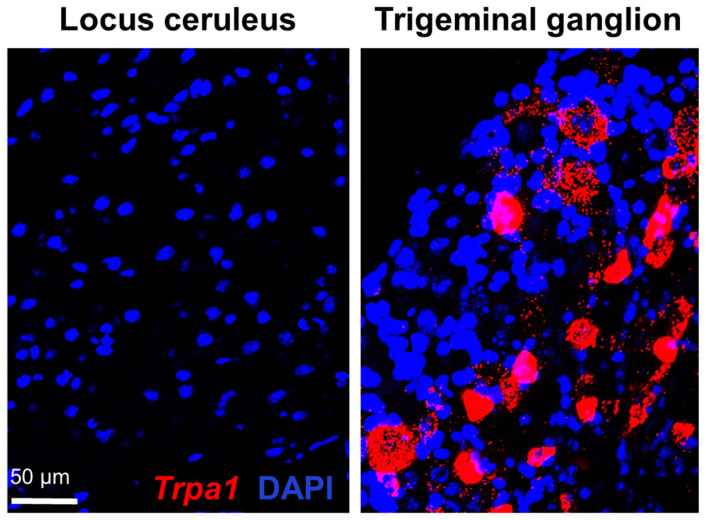
Expression of transient receptor potential ankyrin 1 (*Trpa1*) mRNA (red) in the locus ceruleus and the positive control, trigeminal ganglion, using confocal laser scanning microscopy. The slides were counterstained with the nuclear staining 4′,6-diamidino-2-phenylindole (DAPI, blue).

Confocal laser scanning microscopy and a morphological assessment via RNAscope ISH revealed that no LC neurons express *Trpa1* mRNA. The red fluorophore, Cyanine 3, representing the *Trpa1* mRNA, was completely absent in the LC, but it showed a reliable signal in our positive control, in sections of the trigeminal ganglion (Figure 1).

### 2.2. Comparison of Tyrosine Hydroxylase Content of the Locus Ceruleus in Trpa1 Knockout and Wildtype Mice upon Chronic Stress

**Figure 2:**
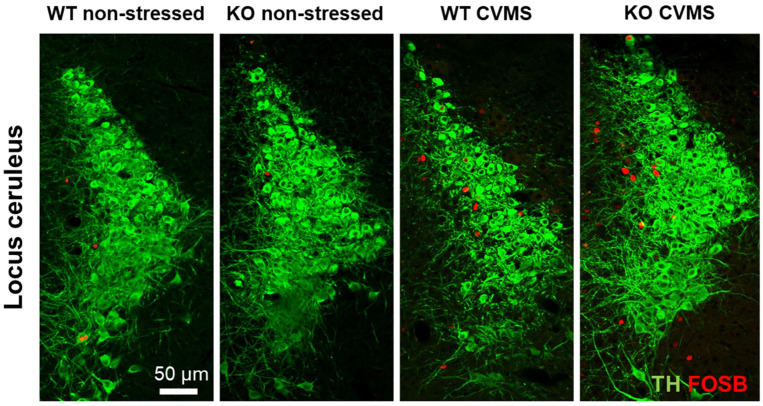
Representative images of tyrosine hydroxylase (TH, green) and FOSB (red) immunofluorescence in the locus ceruleus of *Trpa1* knockout (KO) and wildtype (WT) mice upon chronic variable mild stress (CVMS).

**Figure 3:**
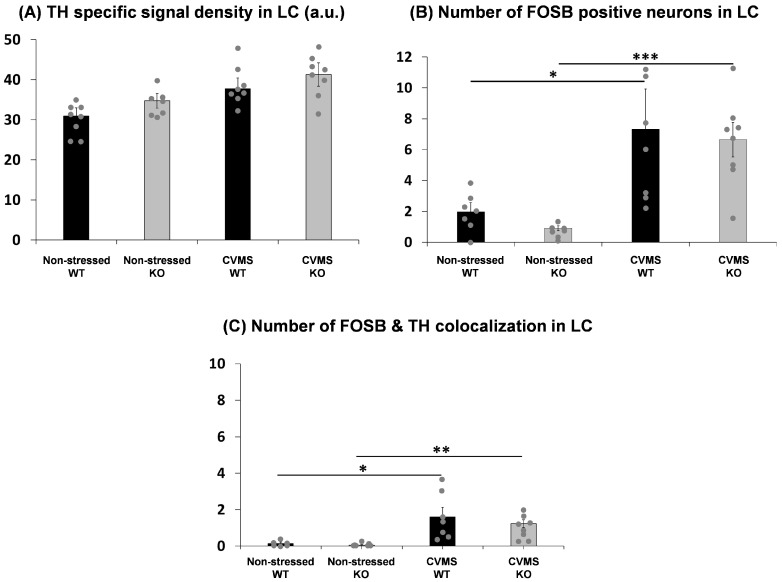
(**A**) Tyrosine hydroxylase (TH) specific signal density (SSD) in the locus ceruleus (LC) upon chronic variable mild stress (CVMS). Columns show the specific signal density in arbitrary units (a.u.). Black bars represent the wildtype (WT) mice, while gray columns refer to the *Trpa1* knockout (KO) mice. Two-way ANOVA followed by Tukey’s post hoc test, n = 7–8/group. (**B**) FOSB neuronal activity in the LC upon CVMS. Columns show the number of FOSB positive neurons. Two-way ANOVA followed by Tukey’s post hoc test, * *p* = 0.01, *** *p* = 0.0005, n = 7–8/group. (**C**) Colocalization of TH and FOSB in the LC upon CVMS. Columns show the number of colocalized neurons. Two-way ANOVA followed by Tukey’s post hoc test, * *p* = 0.01, ** *p* = 0.009, n = 7–8/group.

In order to assess whether CVMS affects the level of TH in the LC, we semi-quantified the TH immunofluorescence (Figure 2). The CVMS had a main effect (F = 6.99; *p* = 0.0155) in the two-way ANOVA, but the post hoc test did not identify any significant differences between the genotypes (Figure 3A).

### 2.3. Evaluation of Chronic Stress-Induced Neuronal Activity in the Locus Ceruleus of Trpa1 Knockout and Wildtype Mice

In order to evaluate the CVMS-induced neuronal activation in the LC, we performed FOSB immunofluorescence staining (Figure 2). CVMS had a main effect (F = 23.02; *p* = 0.00007) in the two-way ANOVA. We observed a marked increase in FOSB positivity upon chronic stress in both WT (*p* = 0.01) and KO (*p* = 0.0005) mice (Figure 3B).

We also counted the number of neurons showing colocalization of TH and FOSB in the LC to determine how many of the chronically active neurons were noradrenergic. However, even though CVMS had a main effect (F = 18.36; *p* = 0.0002) in the two-way ANOVA, we observed that only very few FOSB-positive nuclei (one or two cells *per* section) were localized to TH immunoreactive neurons upon CVMS in both WT (*p* = 0.01) and KO (*p* = 0.009) mice (Figure 3C).

## 3. Discussion

Our research group confirmed the expression of *Trpa1* mRNA in certain stress-related brain areas [10,11,12] in mice and in humans [12]. The most important systems involved in stress adaptation and regulation include the HPA axis, serotonergic and noradrenergic systems [31,32]. In this study, we aimed to test if TRPA1 contributes to the stress adaptation via noradrenergic neurons of the LC. We applied the widely used and well-characterized CVMS model of depression in *Trpa1* KO and WT mice.

For the present study, we used tissue samples from a CVMS study that was recently published [12], and the stress model’s efficacy was confirmed. Shortly, the typical physical (body weight, relative thymus and adrenal gland weight), hormonal (adrenocorticotropic hormone, corticosterone) and behavioral (anxiety, anhedonia, depression-like behavior) indicators of response to CVMS suggested increased HPA axis activity and an elevated depression and anxiety level, confirming the reliability of our model [12].

Even though we found no *Trpa1* mRNA in the LC, the possibility of an indirect effect of this receptor on the noradrenergic system could not be excluded. Therefore, we put forward to examine whether the TRPA1 has any functional significance in the LC in the CVMS-exposed *Trpa1* KO mice vs. non-stressed controls. In contrast to our expectation, no direct or indirect effects of the lack of functional TRPA1 were observed. Neither the FOSB immunoreactivity nor the TH content of the LC neurons were affected by any genotype effect upon chronic stress. The extremely low number of FOSB-TH colocalizations strongly suggests that the CVMS exposure does not force the noradrenergic cells to perform adaptive changes that require the transcription of new genes via activator protein 1 [33]. This, however, does not exclude that alternative signal transduction pathways may have been affected (not examined here). Nevertheless, we must state that our findings ultimately suggest that TRPA1 has no prominent role in the regulation of noradrenergic system in the stress adaptation response. On the other hand, this also suggests that the altered stress response observed in *Trpa1* KO mice should be attributed to another presumably TRPA1-expressing brain region such as the EWcp [12].

The presence [12] and the functional activity [34] of TRPA1 has already been proven in the urocortinergic cells of the EWcp by our research group. Urocortin 1 (UCN1) is a neuropeptide that belongs to the CRH peptide family and has an important role in stress adaptation as it influences the HPA axis. Hyperactivity of the urocortinergic neurons in the EWcp in response to stress has been demonstrated in mice [12], rats [33] and nonhuman primates [35]. Importantly, in humans, a nine-times higher *UCN1* mRNA expression was found in depressed men who died by suicide compared to controls who did not suffer from any central neuropathologies [36]. Most recently, we showed first that *TRPA1* mRNA is expressed also in the human EWcp, and that *TRPA1* is downregulated in samples from male suicide victims [12].

In accordance with the human findings and using the CVMS model of depression in mice, we demonstrated that the UCN1 peptide content was increased while the level of *Trpa1* mRNA was downregulated in EWcp neurons upon stress, further supporting the regulatory role of TRPA1 in stress adaptation [12]. Importantly, the same downregulation of *Trpa1* was observed in the PTSD mouse model [19], supporting the role of TRPA1 in the stress response. Taken together, these findings support the involvement of TRPA1 in stress adaptation linked to EWcp urocortinergic cells.

In our ongoing research, we started to examine the role of the DRN because uro-cortinergic fibers from the EWcp influence both serotonin release and mood status through the CRH receptors of serotonergic cells [37,38]. DR expresses both CRH-R1 and CRH-R2, which convey opposing effects; the CRH-R1 activation results in a reduced serotonin release, while CRH-R2 activation increases it. In resting state, mostly CRH-R1s are present in the membrane of serotonergic cells, while upon chronic stress, the CRH-R2 expression dominates [39].

Further studies are needed to clarify the exact physiological and pathological role of TRPA1 in the central stress response and its potential therapeutic value. Considering the limited distribution of *Trpa1* in the CNS, it is a promising potential drug target with a presumably limited spectrum of central side effects.

## 4. Materials and Methods

### 4.1. Animals

Animals were housed in a temperature- and humidity-controlled environment with a 12 h light–dark cycle (lights on at 6 a.m.) in standard polycarbonate cages (365 mm × 207 mm × 104 mm) in groups of four to six mice *per* cage at the animal facility of the Department of Pharmacology and Pharmacotherapy. Standard rodent chow and tap water were provided ad libitum to the mice. All procedures applied in this protocol were approved by the Ethical Committee on the Use of Laboratory Animals at the University of Pécs (permission No: BA02/2000/33/2018) in agreement with the directive of the European Communities Council in 1986, and with the Law of XXCIII, in 1998, on Animal Care and Use in Hungary.

The original breeding pairs of *Trpa1* KO mice were obtained from Prof. P. Geppetti, University of Florence, Italy. The mice were raised and characterized as described earlier [12]. Animals were bred on C57BL/6J background and crossed back after 10 generations. The offspring were genotyped for the *Trpa1* gene via PCR (sequences of primers: ASM2: ATC ACC TAC CAG TAA GTT CAT; ASP2: AGC TGC ATG TGT GAA TTA AAT).

### 4.2. Experimental Design

Naïve C57BL/6J mice (n = 6) were used to examine the expression of *Trpa1* mRNA in the LC and trigeminal ganglion. In an independent experiment, adult male *Trpa1* KO mice (n = 30) and their WT counterparts (n = 30) were used in the CVMS model of depression. Mice were assigned to four experimental groups: *Trpa1* KO (n = 16) and WT (n = 16) mice that were subjected to CVMS for 21 days, and another set of *Trpa1* KO (n = 14) and WT (n = 14) mice that were not challenged and used as non-stressed controls. Litters of 4–5 different dams were used and offspring were randomly assigned to treatment groups in order to balance litter differences. During the first week, control mice were studied in a behavioral test battery. After that, they were left undisturbed for the last two weeks prior to perfusion to avoid a bias from the stress effect of behavioral testing [12].

### 4.3. Chronic Variable Mild Stress Paradigm

Animals were handled twice a week during a two-week period prior to the CVMS experiment. The three-week CVMS paradigm was applied as published earlier [12]. Briefly, the paradigm involved mid-day (applied between 8 and 11 a.m.) and overnight stressors. For “tilted cage stress”, the box of the mice was fixed in an oblique position of 45°. For “shaker stress”, the mice were placed on an orbital laboratory shaker, which was set to 60 rounds per minute with an orbital diameter of 3 cm. For “restraint stress”, mice were enclosed in a 25 mm diameter plastic tube with a perforated conical tip and several additional ventilation holes for 30 min. The “dark room” exposure was achieved by placing the mice in their home cage in complete darkness for 3 h during the light phase. Following the mid-day stress exposure, animals were placed back to their normal home cages. The overnight stress exposure started at 6 p.m., except on the “group holding” nights during which animals were left undisturbed.

For “social isolation stress”, mice were individually housed overnight, and in the following morning, the original groups were reunited. For the “wet bedding” exposure, the wood shavings were moisturized with 150 mL of tap water in the evening, and the mice were placed on a fresh and dry nesting material in the next morning. Twice a week, the bodyweight of all mice was measured during the time of the regular cage cleaning procedure. Behavioral tests were performed in the CVMS group during the last week, and they were considered mid-day stressors.

### 4.4. Perfusion and Tissue Collection

On day 23, and at least 24 h after any manipulation, the mice were euthanized with an overdose of urethane (2.4 g/Kg), which was administered via intraperitoneal injection. Within 2 min, all mice in the same cage became unconscious. The disappearance of the pain reaction was verified by pinching the tail or paw with forceps. They were then weighed and tail clipped to validate their genotype. After this, the chest cavity was rapidly opened and a needle was introduced through the left ventricle into the proximal ascending aorta. The mice were then perfused with 20 mL of ice-cold 0.1 phosphate-buffered saline (PBS, pH 7.4), followed by 150 mL of a 4% paraformaldehyde (PFA) fixative solution in Millonig buffer (pH 7.4) for 15 min.

After perfusion, the mice were decapitated, and their brains were dissected and post-fixed in the same fixative for 72 h at 4 °C. Using a Leica VT1000S vibratome (Leica Biosystems, Wetzlar, Germany), the brains were coronally sectioned. Four series of 30 µm sections were collected and stored at 4 °C in PBS containing 0.01% sodium azide, until further use. Four representative sections of the LC area (from Bregma −5.34 mm and −5.68 mm according to Paxinos and Franklin, 2001 [40]) per animal were selected for RNAscope ISH and immunofluorescence studies.

### 4.5. RNAscope In Situ Hybridization

The RNAscope ISH [41] was used to detect *Trpa1* mRNA in the LC. The pretreatment procedure was optimized for 30 µm thick PFA-fixed sections. Further steps of the RNAscope protocol (probe hybridization, signal amplification and channel development) were performed according to the RNAscope Multiplex Fluorescent Reagent Kit v2 user’s manual (ACD, Hayward, CA, USA). Briefly, tissue pretreatment was performed using a 1 *v*/*v*% H_2_O_2_ solution in PBS for 30 min. After PBS washes, sections were mounted on Superfrost Ultra Plus slides (Thermo Fisher Scientific, Waltham, MA, USA). The sections were air-dried for 3 h at room temperature (RT) and incubated at 60 °C for 60 min. After 2 × 10 min washes in Milli-Q water (MQ), the slides were incubated in a 10% neutral buffered formalin solution (NBF, Merck KgaA, Rahway, NJ USA) at 4 °C for 2 min. After 3 × 10 min MQ water rinses, the sections were digested in proteinase K solution at 37 °C for 20 min (100 µL of 20 mg/mL proteinase K (EO0491, Thermo Fisher Scientific, Waltham, MA, USA) in a 200 mL buffer (0.1 M Tris/HCl, pH = 8; 0.05 M EDTA, pH = 8). After rinsing in MQ water, the slides were treated with 10% NBF at 4 °C for 2 min, followed by 3 × 10 min washes in MQ water.

Mouse *Trpa1* (ACD, Hayward, CA, USA; Cat. No.: 400211) probes were used and then visualized using cyanine 3 (Cy3) dye (1:750) for the detection of *Trpa1* mRNA in the LC and trigeminal ganglion. After 2 × 15 min washes with PBS, the sections were counterstained with 4′,6-diamidino-2-phenylindole (DAPI) (ACD, Hayward, CA, USA). Finally, after a PBS wash, the slides were covered with ProLong Gold Antifade (Thermo Fisher Scientific, Waltham, MA, USA; Cat. No.: P10144) mounting medium. After drying at 4 °C, the sections were stored at −20 °C until digitalization.

As a positive control, a mouse trigeminal ganglion was used, which is known for its high *Trpa1* expression. Moreover, mouse (ACD, Hayward, CA, USA; Cat. No.: 320881) triplex positive control and triplex negative (ACD, Hayward, CA, USA; Cat. No.: 320871) control probes were tested on the LC samples. The triplex positive control probes exhibited a well-detectable signal, while the negative control probes did not exhibit any recognizable fluorescence in the preparations.

### 4.6. Tyrosine Hydroxylase and FOSB Double Immunofluorescence

Sections were washed in PBS 3 × 10 min on a filter shaker. To accomplish the disruption of the cell membranes, the sections were treated in 0.5% Triton X-100 (Sigma-Aldrich, St. Louis, MO, USA) detergent with PBS for 30 min on a filter shaker. To block non-specific binding sites, the sections were treated in 2% normal donkey serum (NDS) with PBS on shaker for 30 min. The slides were placed in 2% NDS in PBS containing primary antibodies: mouse anti-TH (Sigma-Aldrich, St. Louis, MO, USA, Cat. No.: T2928, in 1:1000 dilution) and rabbit anti-FOSB (Abcam, Cambridge, UK, Cat. No.: ab184938, in 1:6000 dilution) antibodies at RT overnight.

The following day, the sections were washed for 3 × 10 min in PBS and incubated for 3 h with the secondary antibodies (Alexa 488-conjugated donkey anti-mouse (Jackson Immunoresearch Europe Ltd, Ely, UK, Cat. No.: 715-545-150, in 1:500 dilution) and Cy3-conjugated donkey anti-rabbit (Jackson Immunoresearch Europe Ltd, Ely, UK, Cat. No.: 711-165-152, in 1:500 dilution) dissolved in PBS and 2% NDS. After 3 × 10 min washes in PBS, the sections were placed on gelatin-coated slides and dried for 1 h at RT in the dark, after which, they were covered with glycerol-PBS (1:1) and stored at −20 °C until the microscopic examination.

### 4.7. Microscopy, Digital Imaging and Morphometry

Microscopic preparations were examined using an Olympus FluoView 1000 confocal microscope (Olympus, Hamburg, Germany). Digital images were acquired by sequentially scanning in analogue mode for the respective fluorophores in order to avoid false positive signals due to the slightly overlapping emission spectra, and to detect reliably quantifiable fluorescent signals. For scanning, a confocal aperture of 80 µm was used, with 1024 × 1024-pixel resolution and a 40× objective. The optical thickness of the digital scans was 3.5 µm. The excitation and emission spectra for the respective fluorophores were selected by applying the built-in settings of the FluoView software (FV10-ASW Version 0102, Olympus, Hamburg). Alexa 488 was excited at 488 nm, Cy3 at 550 nm and DAPI at 405 nm.

Sections were scanned for the respective wavelengths at three channels. Digital scan images of the three channels, showing the same area, were automatically superimposed and merged. The colocalization was evaluated on digital images showing virtual green (Alexa 488) and red (Cy3) colors, representing the fluorescent signals of the two channels.

The intensity of the fluorescence for TH was measured using Image J software (version 1.42., NIH, Bethesda, MD, USA), using four non-edited images of the corresponding channel. The region of interest was manually determined at cytoplasmic areas of neurons, without marking the area of the cell nucleus. The signal area measured here was then corrected for the background signal. The specific signal density (SSD) was expressed in arbitrary units (a.u.). The average of the SSD of neurons was calculated in four sections *per* animal. The average of these four values represented the SSD value of one mouse.

In case of FOSB immunofluorescence, the number of FOSB positive neurons in one section was counted manually, in four representative sections *per* animal. Finally, these values were averaged as described above, and thereafter subjected to statistical assessment.

For the evaluation of FOSB and TH colocalization, the neurons showing both red (FOSB) and green (TH) signals were counted manually on each slide.

### 4.8. Statistics

For each experimental group, the data were expressed as mean ± standard error of the mean. The datasets were tested for normality using the Shapiro–Wilk test [42], and for homogeneity of variance using Barlett’s chi-square test [43]. Outlier data beyond the two-sigma range were excluded from the statistics.

The data were assessed via two-way analysis of variance (ANOVA, factors: stress and genotype), followed by Tukey’s post hoc test. The post hoc tests were performed based on the first- or second-order effects in the ANOVA tests. The analyses were performed using Statistica 8.0 (StatSoft, Tulsa, OK, USA) software (alpha = 5%).

## 5. Conclusions

There is no *Trpa1* mRNA expression in the LC, and a lack of TRPA1 has neither a direct nor indirect effect on the LC noradrenergic neurons upon chronic stress. These results further support the involvement of EWcp/TRPA1 in stress adaptation.

## Data Availability

The datasets used and/or analyzed during the current study are available from the corresponding author upon reasonable request.
